# Human Joint Angle Estimation Using Deep Learning-Based Three-Dimensional Human Pose Estimation for Application in a Real Environment

**DOI:** 10.3390/s24123823

**Published:** 2024-06-13

**Authors:** Jin-Young Choi, Eunju Ha, Minji Son, Jean-Hong Jeon, Jong-Wook Kim

**Affiliations:** 1 Department of Electronic Engineering, Seunghak Campus, Dong-A University, Busan 49315, Republic of Korea; jinyoung0725@gmail.com (J.-Y.C.); 19731jmj@naver.com (E.H.); 2 Research Institute, JEIOS Inc., Busan 46903, Republic of Korea; son@jeios.com (M.S.); jeanhongjeon@gmail.com (J.-H.J.)

**Keywords:** human pose estimation, monocular camera, image processing, optimization, humanoid model

## Abstract

Human pose estimation (HPE) is a technique used in computer vision and artificial intelligence to detect and track human body parts and poses using images or videos. Widely used in augmented reality, animation, fitness applications, and surveillance, HPE methods that employ monocular cameras are highly versatile and applicable to standard videos and CCTV footage. These methods have evolved from two-dimensional (2D) to three-dimensional (3D) pose estimation. However, in real-world environments, current 3D HPE methods trained on laboratory-based motion capture data encounter challenges, such as limited training data, depth ambiguity, left/right switching, and issues with occlusions. In this study, four 3D HPE methods were compared based on their strengths and weaknesses using real-world videos. Joint position correction techniques were proposed to eliminate and correct anomalies such as left/right inversion and false detections of joint positions in daily life motions. Joint angle trajectories were obtained for intuitive and informative human activity recognition using an optimization method based on a 3D humanoid simulator, with the joint position corrected by the proposed technique as the input. The efficacy of the proposed method was verified by applying it to three types of freehand gymnastic exercises and comparing the joint angle trajectories during motion.

## 1. Introduction

The field of 3D motion analysis is rapidly evolving, particularly in sports, home fitness, and healthcare. Consequently, several advanced technologies are emerging in the market. According to the 2022 survey, the global 3D motion capture market is expected to generate USD 1.165 billion by 2033 [1].

There are two clear divisions in motion-capture technology: (i) marker/optical systems that often use infrared cameras and reflective markers and (ii) marker-less motion-capture (MLMC) systems, which are growing in popularity because of their lower costs and ease of use in less complex tasks, such as treadmill analysis during running. In contrast to traditional motion analyses, an MLMC system does not require markers on the body, thereby simplifying the process significantly. In particular, it can be utilized to identify neurological conditions, such as Parkinson’s disease, by analyzing the body’s walking patterns or gait [2]. An alternative method for motion capture involves the use of inertial measurement units (IMU) that encompass accelerometers and gyroscopes [3]. Although these sensors do not offer exhaustive data capture for full-body systems, they can effectively capture motion to a significant degree.

Motion-capture technology is widely used for gait analysis in sports and is essential for activities involving running motion, such as sports medicine, to study athletic movements and identify dysfunctions related to injuries [4]. This technology is crucial for understanding athlete success and handling complex injuries. Moreover, MLMC technology has been tested in a community setting and has proven particularly useful for the identification of neurological impairments and tracking rehabilitation progress.

Accordingly, the technology for human pose estimation (HPE) using monocular camera sensors has witnessed rapid development. Monocular HPE is used to locate the 3D positions of human body joints in 2D images or videos. The existing studies can be divided into two categories: deterministic and probabilistic. The deterministic approaches in [5,6,7] produced a single definite 3D pose for each image, whereas the probabilistic approaches in [8,9,10] represented 2D to 3D lifting as a probability distribution and produced a set of possible solutions for each image. In [11], both approaches were combined by aggregating multiple-pose hypotheses into single and higher-quality 3D poses. The deterministic approach is more practical for real-world applications; thus, they are suitable for real-time HPE. Deterministic approaches rely on pixel-aligned 3D keypoints [6], mesh vertices [12], and mesh-aligned features [13] to obtain accurate HPE. Among these, pixel-aligned approaches exhibit high HPE accuracy; however, in deep learning (DL) methods, various challenges remain, including occluded areas and a lack of training data [14,15]. To address these issues, a method has been proposed to alleviate the occlusion problem using sensor fusion [16] and multiple cameras [17]. However, its application in real-world environments remains challenging.

Although a previous study [18] comprehensively evaluated the performance of the latest 3D HPE algorithms, the performance evaluation of inference accuracy issues and inference time for previously known problems, such as occlusion, remains unclear. In this study, we focused on single-view single-person 3D HPE to identify problems when applied to motion recognition in various real-world videos using four 3D HPE methods: MediaPipe Pose (MPP) [5], Hybrid Inverse Kinematics solution (HybrIK) [6], Multi-Hypothesis Transformer (MHFormer) [10], and Diffusion-based 3D Pose Estimation (D3DP) [11] to analyze the problem. In addition, we proposed data-processing techniques to eliminate and correct anomalies, such as left/right joint position inversion and false detections in daily life motions. Finally, joint angle trajectories of a 3D humanoid simulator were obtained for intuitive and informative human activity recognition (HAR) using the univariate dynamic encoding algorithm for searches (uDEAS), which has been proven to be successful for 2D joint coordinates [19,20]. Used as input, the 3D joint coordinate data were corrected by applying the proposed data-correction technique. If the accuracy of joint angle-based 3D HAR using a monocular camera becomes acceptable, it can be applied to a wide range of fields, such as recognizing hazardous behaviors in daily life, autonomous driving, personalized home care, metaverse, healthcare, and medical clinical rehabilitation therapy.

## 2. Related Work

**Figure 1:**
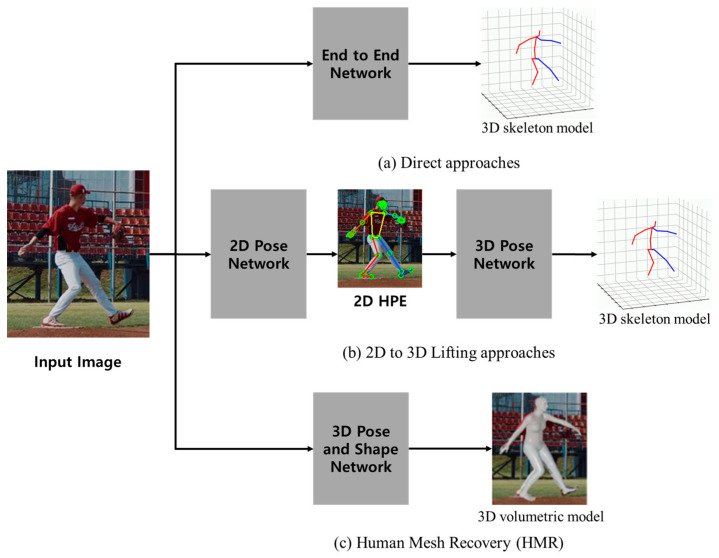
The 3D HPE frameworks: (**a**) skeleton model—direct approaches; (**b**) skeleton model—2D to 3D lifting approaches; (**c**) volumetric model—human mesh recovery [18].

According to recent research, the 3D HPE approach determines whether to reconstruct only the skeleton or recover the 3D human mesh using a skeleton and volumetric model [18]. Figure 1 shows the 3D HPE framework configuration diagram commonly used in a single-view, single-person approach.

### 2.1. Skeleton Model

The human skeleton model is advantageous because it intuitively describes the structure of the human body using a tree structure that links the joints with lines. This model is used not only for 3D pose estimation but also for 2D pose estimation because of its simple structure, which reduces the computational cost and time. Previous studies can be classified into direct estimation and 2D to 3D lifting approaches.

The direct estimation approach involves a single step; it directly infers 3D joint locations from images or videos via an end-to-end network. A representative algorithm is MPP, an open-source library released by Google in 2020. MPP estimates 33 landmarks of the human body joints using the BlazePose model [21]. Research analyzing motions in activities of daily living [19,20] and karate using MPP has recently gained momentum [22]. MPP uses a detector-tracker ML pipeline. First, a pose detector identifies the region of interest (ROI) within an RGB image using facial landmarks to determine the presence of a person. Subsequently, a pose tracker infers 33 landmarks within the ROI.

The 2D to 3D lifting approach comprises two steps. It estimates the 2D pose from the input images or videos and then the 3D joint locations. Representative approaches for 2D to 3D lifting approach include transformer-based and diffusion-based approaches. Diffusion models generate high-dimensional data through the gradual transformation of the data. This is a D3DP diffusion-based 3D HPE method proposed in 2023. First, the D3DP generates multiple possible 3D pose hypotheses for a single 2D observation. Second, it gradually diffuses the ground-truth 3D poses into a random distribution and learns a denoiser conditioned on 2D keypoints to recover the uncontaminated 3D poses. Third, joint-wise reproduction-based multi-hypothesis aggregation (JPMA) is used to combine multiple generated hypotheses into a single 3D pose. Consequently, it reprojects 3D pose hypotheses onto a 2D camera plane, selects the best hypothesis joint-by-joint based on reprojection errors, and combines the selected joints into the final pose [11]. The transformer architecture, originally used primarily in natural language processing, is now also being applied in the field of computer vision. It learns the relationships between tokens extracted from images to infer 3D poses. In MHFormer, proposed in 2022, multi-hypothesis spatiotemporal feature structures are explicitly combined into transformer models, and the multiple hypotheses of body joint information attained in 2D to 3D lifting are independently and mutually processed in an end-to-end manner. The MHFormer is decomposed into three stages. First, multiple initial hypothesis representations are generated. Second, for model self-hypothesis communication, multiple hypotheses are merged into a single converged representation and then partitioned into several divergent hypotheses. Third, cross-hypothesis communication is learned, and multi-hypothesis features are aggregated to synthesize the final 3D pose [10].

### 2.2. Volumetric Model

The human mesh recovery (HMR) technique, which represents the human body in a 3D mesh form from a single image, has gained attention in recent developments. This method involves reconstructing the human body as a 3D volumetric mesh model using an input image or video. A notable 3D mesh model used in this context is the skinned multiperson linear (SMPL) model [23]. DL algorithms based on a 3D mesh model demonstrate improved accuracy in pose estimation by considering the body shape and rotation matrices, thus accounting for twisting movements. However, these algorithms incur high computational costs and long processing times. In addition, the limitations of the 3D joint coordinate datasets used for DL training often result in lower accuracy for untrained poses. Training on datasets containing 3D information typically involves capturing data in laboratory settings using motion-capture equipment. This can increase the likelihood of false detections in clothing and real-world environments. Previous studies based on HMR included Pose2Pose [24], HybrIK [6], and FrankMoCap [25]. These research efforts reflect the ongoing challenges and developments in the field of 3D human pose estimation. HybrIK, proposed in 2020, is an inverse kinematics solution that considers the volume of a human body in 3D. Previous estimation methods based on HMR reconstructed a 3D mesh by estimating multiple parameters. However, the learning of abstract parameters can degrade the model’s performance. Thus, HybrIK employs an inverse kinematics approach to bridge the gap between mesh and 3D skeletal coordinate estimation. It supports two models: SMPL [23] and SMPL-X [26].

## 3. Methodology

### 3.1. Pose-Estimation Methods

**Figure 2:**
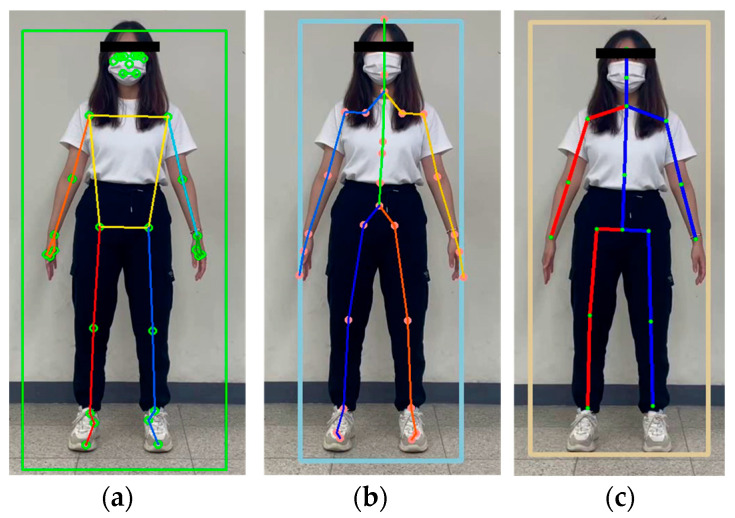
HPE landmarks: (**a**) 33 landmarks of MPP; (**b**) 29 landmarks of HybrIK; and (**c**) 17 landmarks of MHFormer and D3DP.

In this study, we implemented and compared the end-to-end deep learning models MPP and HybrIK and the hybrid models MHFormer and D3DP. The implementation environments for these methods are presented in Table 1. In addition, Figure 2 shows the landmark locations for each algorithm.

### 3.2. Performance Comparison in Real-World Environments

To compare the accuracy of the DL models in real-world environments, we conducted a comparison using video footage. Specifically, RGB videos recorded at a resolution of 1280 × 720 pixels and 30 FPS were used as inputs for the DL models. The selected input videos included complex postures, scenes with objects resembling human figures, footage recorded from a distance, and videos captured under various lighting conditions. This section analyzes the limitations and challenges of deep-learning models in real-world environments.

**Figure 3:**
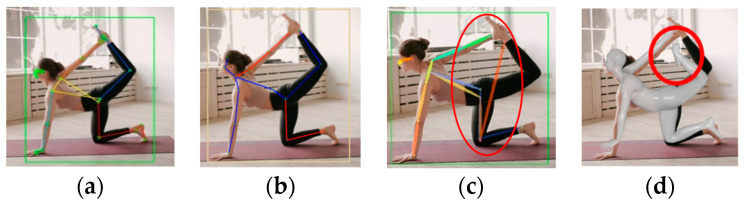
Estimation results of a yoga pose: (**a**) MPP; (**b**) MHFormer; (**c**) D3DP; and (**d**) HybrIK (square: BB indicating human recognition area, red circle: inaccurate estimation).

Figure 3 shows the first video, featuring a complex yoga pose with intertwined human joints. In Figure 3a,b, the skeleton models estimated using MPP and MHFormer were overlaid. The area wherein a person was recognized was marked using bounding boxes (BBs). Even for a single image, the model accurately detected the upward bending of the left leg. This demonstrates the effectiveness of the MPP and MHFormer in complex pose recognition. Figure 3c,d show the estimation results in which the skeletal model, estimated to be D3DP, and the SMPL model, estimated to be HybrIK, were overlaid on the image. The algorithm accurately estimates the leg positions within a certain angle range. However, as the complexity of the pose increased, the estimation accuracy decreased.

**Figure 4:**
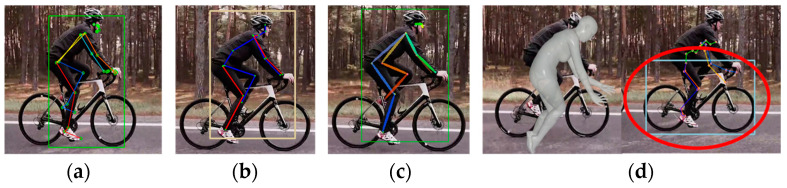
Estimation results of videos including objects: (**a**) MPP; (**b**) MHFormer; (**c**) D3DP; and (**d**) HybrIK (square: BB indicating human recognition area, red circle: inaccurate estimation).

Next, we compared the estimation results for a person riding a bicycle. Figure 4 shows an image of a person cycling shot from the side, which includes occlusion areas where certain joints were obscured and external objects were present. In Figure 4a–c, MPP, MHFormer, and D3DP accurately identified the joints of a person without mistaking the bicycle as a human figure, respectively. However, as shown in Figure 4d, HybrIK attempted to misidentify a bicyclist as a person and estimate the 3D posture. Thus, the estimated SMPL model deviated significantly from that of the target person.

**Figure 5:**
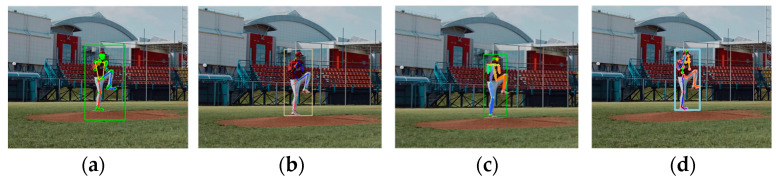
Estimation results for long-distance videos: (**a**) MPP; (**b**) MHFormer; (**c**) D3DP; and (**d**) HybrIK (square: BB indicating human recognition area).

The third video was shot from a distance and featured an individual in a pitching stance. Owing to the camera angle, certain joints of the person were located in self-occluded areas. Figure 5a–d present the estimation results for MPP, MHFormer, D3DP, and HybrIK, respectively. The four methods demonstrated good estimation accuracy for this scenario, indicating their effectiveness in addressing challenges such as distant subjects, particularly in capturing and analyzing the posture of a person engaged in a specific activity such as pitching.

**Figure 6:**
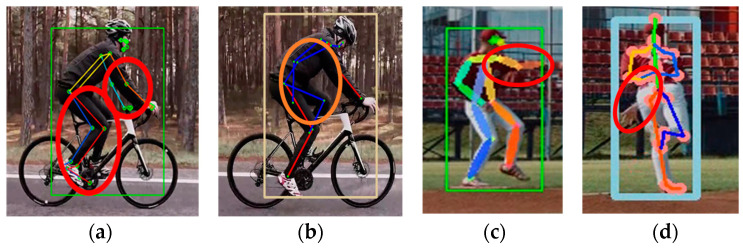
Inaccurate estimation results for occluded areas: (**a**) MPP; (**b**) MHFormer; (**c**) D3DP; and (**d**) HybrIK (square: BB indicating human recognition area, red circle: inaccurate estimation).

However, all four methods yielded unsatisfactory results for occluded areas. Inaccurate estimates of the occluded areas were observed in certain frames, as shown in Figure 6.

The final case involved a video with varying light intensities because of shadows. In real-world environments, sunlight or artificial lighting can cause shadows, and people often wear clothing with various patterns. This can result in frequent and abrupt changes in the color of RGB images.

**Figure 7:**
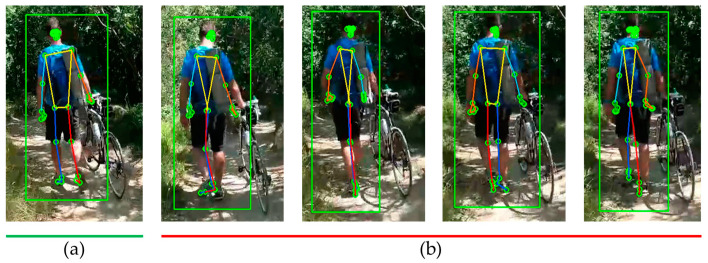
Estimation results of MPP on videos with changing light intensity: (**a**) accurate estimation; (**b**) inaccurate estimation (square: BB indicating human recognition area).

Figure 7 presents the estimation results of the MPP, which demonstrates its capability to estimate poses, even from the back of a person. However, there are frequent occurrences of coordinate inversions on the left and right sides resulting from changes in lighting conditions.

**Figure 8:**
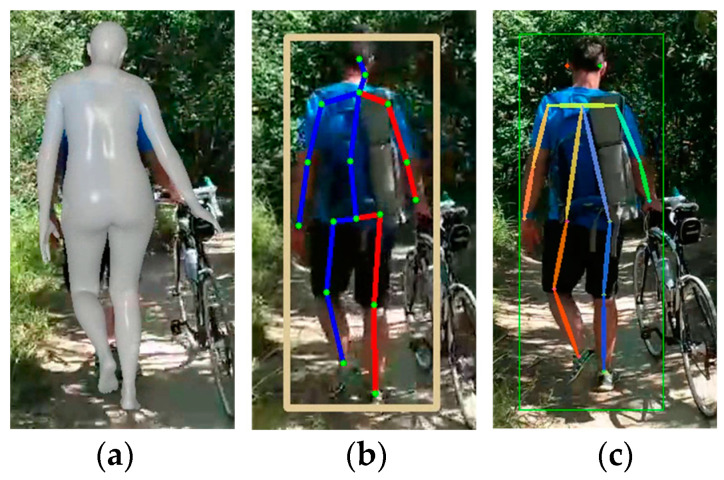
Estimation results for videos with changing light intensity: (**a**) HybrIK with SMPL; (**b**) MHFormer; and (**c**) D3DP (square: BB indicating human recognition area).

Figure 8 presents the estimation results for HybrIK, MHFormer, and D3DP. Similar to the MPP, all methods accurately estimated the pose of a person from the back. Furthermore, it was more robust in handling changes in light intensity compared to the MPP, providing more stable estimation results under varying lighting conditions. This indicated a certain level of resilience to environmental lighting changes, which is crucial for practical applications in diverse real-world scenarios.

Summarizing the results thus far in real-world environments, each DL HPE algorithm generally performed well in human recognition and joint detection. However, in real-world environments, we observed inaccurate estimation results in certain frames when various objects, light changes, or occlusions were present. In particular, a decrease in joint position estimation accuracy in complex intertwined postures, such as yoga postures, was observed. Furthermore, in the case of MPP, a left-right switching phenomenon was observed, and HybrIK showed a decrease in joint detection accuracy owing to human recognition errors.

Finally, it is noteworthy that the four HPE methods did not produce joint angles. To move beyond person recognition from 2D images to recognizing and predicting 3D human actions, accurate estimation of joint angles is essential. Therefore, this study aimed to further investigate the removal and correction of anomalies in DL models to develop a method that would improve the accuracy and applicability of HPE using an optimization method that determines the angles of each joint with reference to a 3D humanoid model.

### 3.3. Improving Pose Recognition Performance

#### 3.3.1. Improving Human Recognition Accuracy

Improving the human recognition performance necessitates the enhancement in the accuracy of DL models. In real-world environments, video data often contain multiple people and objects resembling human figures. Thus, for 3D HPE technology that focuses on a single person to be effectively utilized in real environments, it is crucial that the individual in a video is continuously recognized.

A comparative study revealed instances wherein non-human objects were mistakenly recognized as humans. Originally, HybrIK utilized the fasterrcnn_resnet50_fpn algorithm [27] provided by PyTorch for rapid object detection in images, and the detected region of interest was then input into the HybrIK model. However, this method only estimates the object with the highest recognition score and largest area among those recognized in the image without specifically focusing on human figures. Consequently, non-human objects, such as bicycles, are misidentified as humans, resulting in inaccurate estimations using the HybrIK model. In this study, to improve the recognition accuracy, fasterrcnn_resnet50_fpn trained on the COCO dataset was applied to HybrIK. We used the 2017 version of the COCO dataset [28]. Excluding the background, 11 of the 91 categories in the dataset were omitted, and the classification was conducted on 80 objects. This enhancement aimed to refine the object-detection process by focusing specifically on human figures and reducing the likelihood of misidentifying nonhuman objects as people.

First, the target person for the analysis was identified in the first frame. The object recognition algorithm predicted significantly more BB than the actual objects. Therefore, to adopt the most accurate BB for human recognition, the following steps were performed to eliminate unnecessary BBs.

1. All BBs with confidence scores below a certain threshold were removed.
  (1) $${\mathrm{BB}}_{\mathrm{remove}}=\left\{{\mathrm{BB}}_{i}|{\mathrm{Conf}}_{i}<{\mathrm{Conf}}_{\mathrm{threshold}}\right\}$$
  where ${\mathrm{Conf}}_{i}$ is the confidence score associated with BB i.
2. All detected BBs that were not identified as humans were removed.
  (2) $${\mathrm{BB}}_{\mathrm{human}}=\{{\mathrm{BB}}_{i}|{\mathrm{BB}}_{i}\;\mathrm{is}\;\mathrm{dentified}\;\mathrm{as}\;a\;\mathrm{human}\}$$
3. Finally, the ROI to be analyzed was determined. Among the remaining BBs, only the one with the largest area was retained, and the rest were removed.

If there were no BBs with confidence scores above the threshold in the first frame, the threshold was adjusted and the process was repeated. This approach ensured that the most probable human figure was selected for analysis, thereby enhancing the accuracy of subsequent pose estimation.

(3) $$\mathrm{ROI}={\mathrm{BB}}_{\mathrm{selected}}={\mathrm{argmax}}_{{\mathrm{BB}}_{i}}\left({\mathrm{Area}}_{i}\right),\;{\mathrm{BB}}_{i}\in {\mathrm{BB}}_{\mathrm{human}}$$

Once the subject for analysis was determined, the information from the previous frame was used to continuously recognize the target. The process is as follows.

1. In the current frame, all BBs that were not identified as humans were removed;
2. The intersection over union (IoU) between the BB recognized in the previous frame and BBs in the current frame was calculated. The IoU is a common measure used in object detection to assess the similarity between two sets. This is calculated as the ratio of the intersection area (${\mathrm{bboxArea}}_{\mathrm{inter}}$) of the recognized regions in the current (${\mathrm{bboxArea}}_{\mathrm{cur}}$) and previous (${\mathrm{bboxArea}}_{\mathrm{prev}}$) frames to their union areas, as expressed below:
  (4) $$\mathrm{IoU}\left({\mathrm{bbox}}_{\mathrm{prev}},\;{\mathrm{bbox}}_{\mathrm{cur}}\right)=\frac{{\mathrm{bboxArea}}_{\mathrm{inter}}}{{\mathrm{bboxArea}}_{\mathrm{prev}}+{\mathrm{bboxArea}}_{\mathrm{cur}}-{\mathrm{bboxArea}}_{\mathrm{inter}}}$$
3. Finally, the BB with the highest sum of the confidence and IoU scores was adopted.

This approach ensured continuous and accurate tracking of the target person across frames, leveraging the similarity of the detected regions between consecutive frames and the detection reliability.

(5) $$\mathrm{ROI}={\mathrm{BB}}_{\mathrm{selected}}={\mathrm{argmax}}_{{\mathrm{BB}}_{i}}\left({\mathrm{Conf}}_{i}+{\mathrm{IoU}}_{\max}\right),\;{\mathrm{BB}}_{i}\in {\mathrm{BB}}_{\mathrm{human}}$$

where ${\mathrm{IoU}}_{\max}=\max_{i}\left(\mathrm{IoU}\left({\mathrm{BB}}_{\mathrm{prev}},\;{\mathrm{BB}}_{i}\right)\right)$.

**Figure 9:**
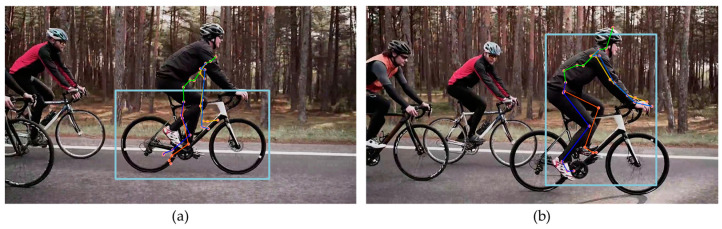
Estimated recognition results in HybrIK compared with proposed human recognition algorithms: (**a**) Before processing; and (**b**) after processing (square: BB indicating human recognition area).

Figure 9 illustrates the results of applying the proposed human recognition algorithm to the HybrIK model, which improved the accuracy of human pose estimation compared with previous results.

#### 3.3.2. Detection of Outliers

In real-world environments, the issues commonly encountered in human model recognition can be categorized as jitter, switching, and misdetection [15]. Therefore, the detection and correction of outliers are essential. In this study, a 3D joint coordinate correction step was conducted to address the shortcomings typically associated with DL-based human pose-estimation algorithms and to improve accuracy.

When capturing movements using a single monocular camera, areas of occlusion occur because of the fixed field of view. These occlusion areas can be categorized as self-occlusions, wherein certain joints are obscured by the body, or external occlusions, wherein the joints are obscured by external objects. Capturing the same pose with different camera positions obscures different joints. In these occluded areas, low-confidence estimates are often generated using DL models.

Another challenging factor in pose estimation from RGB images is the variation in lighting intensity. Irregular changes in lighting can lead to left or right inversions or sudden coordinate distortions. In this study, inaccurate estimations of occlusion areas commonly encountered with DL models and the phenomenon of left or right switching were defined as outliers, and detection and correction were performed. Mis-detection in occluded areas mostly involves the inclusion of end joints.

**Figure 10:**
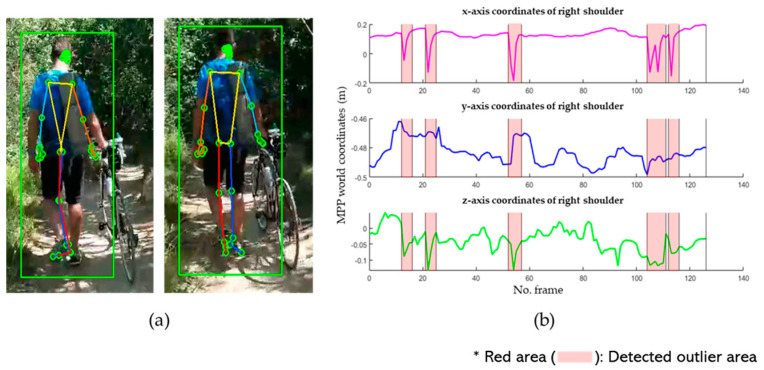
Estimation results of MPP on videos with outliers: (**a**) example image with outliers; and (**b**) 3D MPP coordinate trajectory of the right shoulder (green square: BB indicating human recognition area).

Symmetrical inversion of the shape of the human body occurs when the left and right sides are switched, often around the center points of the pelvis or the joints at the centers of the shoulders and pelvis. Figure 10 illustrates the 3D coordinate trajectory of the right shoulder with outliers, with the segments affected by the outliers marked in red area. The 3D coordinate trajectory was extracted according to the world coordinates provided by MPP [5]. These coordinates were normalized to meters from the center of the hip to the origin. In such cases, all joints must be adjusted because they can occur across the entire body.

In this study, outliers were detected through changes in the lengths of 10 major links, including the shoulder, pelvis, thigh, shin, upper arm, and lower arm. The link length was calculated as the Euclidean distance between two joints in a 3D pixel coordinate system. The measured link lengths in a pixel frame differed depending on the distance from the camera. Therefore, through the key information measured in the pixel image, the link length measured in the pixel coordinate system was converted into centimeters. At this time, the average height information of Size Korea [29] was used to normalize the average height of women and men, 160 and 175 cm.

**Figure 11:**
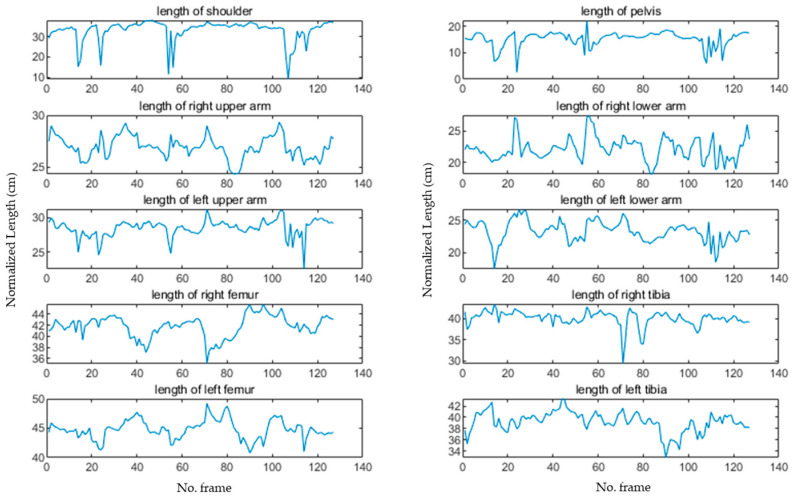
Lengths of major links.

**Figure 12:**
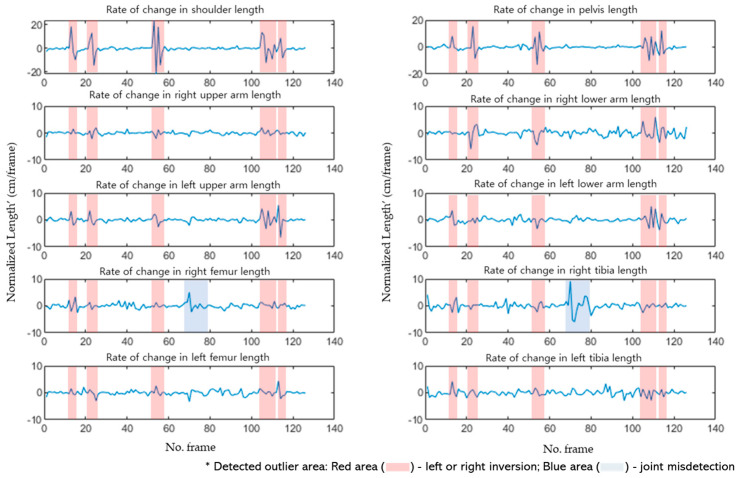
Differentiation results of the lengths of major links.

Figure 11 illustrates the changes in the lengths of the 10 links for each frame during the walking motion, as shown in Figure 10. Joint lengths vary linearly when a person performs dynamic motion. The proposed algorithm differentiated the length changes per frame and detected nonlinear segments. Figure 12 presents the results of differentiating the lengths of the 10 links shown in Figure 11, where the red areas indicate cases of left or right inversion, and the blue areas represent instances of partial joint misdetection.

#### 3.3.3. Outlier Correction

**Figure 13:**
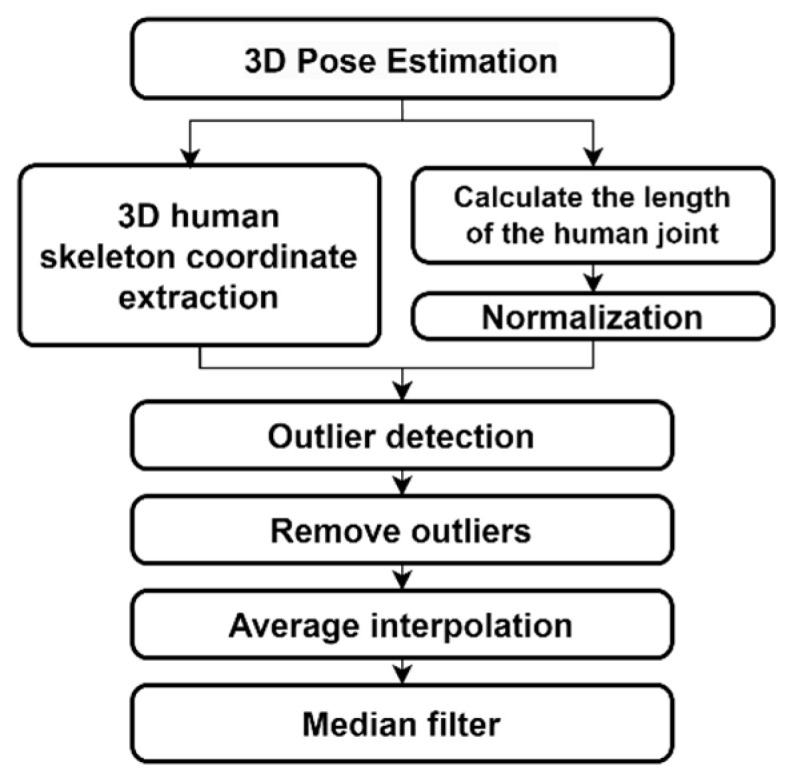
Structure of the proposed outlier detection and correction algorithm.

In this study, we proposed an outlier detection and correction method, the structure of which is shown in Figure 13, using the lengths of the human body links.

**Figure 14:**
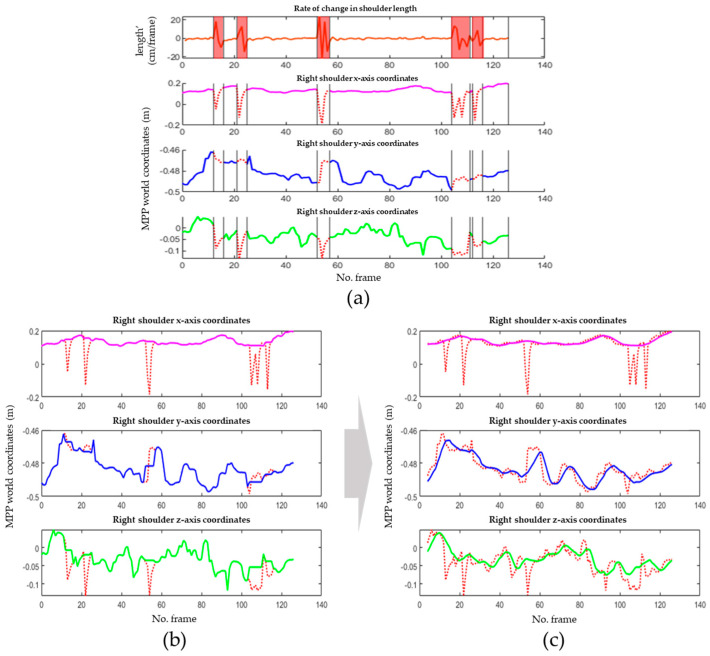
Outlier correction results for the right shoulder joint positions: (**a**) removing outliers; (**b**) average interpolation; and (**c**) median filter (solid line: corrected data, dashed red line: original data, red area: detected outlier area).

Figure 14 shows the outlier correction process for the 3D coordinates of the right shoulder. First, variations in the lengths of the major links were analyzed to observe any changes. Figure 14a shows the length variations of the right shoulder used to detect outliers. Segments with detected outliers are marked in red. The solid lines in Figure 14b,c represent the corrected data after outlier removal, whereas the dashed red line represents the original data. In Figure 14b, the removed segments are interpolated employing the mean interpolation method, using the average values of the frames before and after the outlier segments. As shown in Figure 14c, a median filter was applied to smooth the corrected trajectories. The application of the filter minimized the frame-by-frame errors in the DL model.

#### 3.3.4. Joint Angle Estimation

To estimate the joint angle trajectories of human motion, a 3D humanoid robot model and an optimization algorithm were employed using the joint coordinate trajectories corrected using the data-processing algorithm described previously. The uDEAS was selected as the optimization method because of its high speed and accuracy, as proven in a previous study [30], and the modified version of combinatorial DEAS (cDEAS), which can seek integer variables as well [31].

uDEAS is a global optimization method that combines local and global search schemes by representing real numbers in binary matrices using the decoding function in [31]. In the local search, a session comprising a single bisectional search (BSS) and multiple unidirectional searches (UDS) is sequentially executed for each row from the first to the last variable. The BSS adds a new bit at the rightmost position, and the UDS increases or decreases each binary row (the encoded representation for each variable) depending on the BSS result. With respect to the global optimization scheme, the uDEAS restarts the local search procedure using random binary matrices. Among the local minima identified, the minimum cost function was selected as the global minimum.

As the number of optimization variables increases, searching for them sequentially in a predetermined order in a local search becomes less efficient. To address this, we proposed an adaptive variable-ordering strategy for the uDEAS that prioritized the exploration of variables based on their sensitivity to the cost function. To this end, the cost-sensitivity function of the *i*th variable in the *j*th session, $v_{i}^{j}$, was designed as follows:

(6) $$S(v_{i}^{j})=\frac{\left|\frac{L\left(v_{i,BSS}^{j,l}\right)-L\left(v_{i,BSS}^{j,r}\right)}{v_{i,BSS}^{j,l}-v_{i,BSS}^{j,r}}\right|+\sum_{k=1}^{M}\left|\frac{L\left(v_{i,UDS}^{j,k-1}\right)-L\left(v_{i,UDS}^{j,k}\right)}{v_{i,UDS}^{j,k-1}-v_{i,UDS}^{j,k}}\right|}{M+1}$$

where L is the cost function, $v_{i,BSS}^{j,l/r}$ and $v_{i,UDS}^{j,k}$ denote $v_{i}^{j}$ at the left or right BSS and *k*th iteration of the UDS, respectively, and M is the number of successful UDS iterations following which the cost no longer decreases.

**Figure 15:**
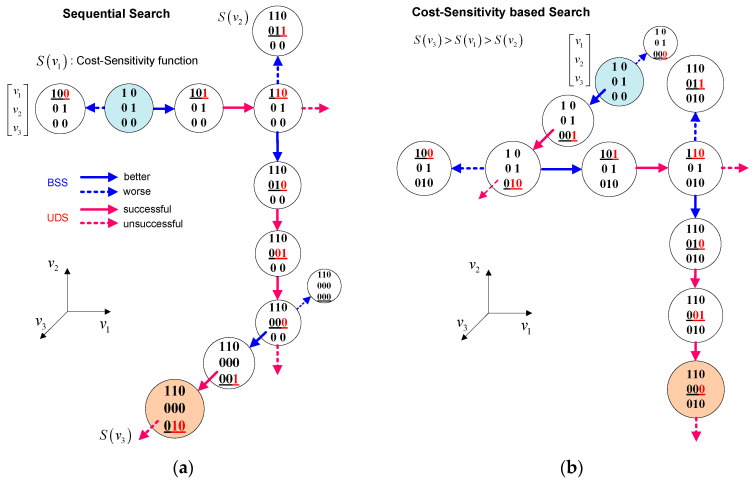
Local search schemes of uDEAS in 3-dimensional search space: (**a**) sequential search; and (**b**) cost-sensitivity based search. (underlined number: modified row, red number: added or modified bit, light blue circle: initial matrix of the session, orange circle: final matrix of the session).

Figure 15a shows an example of a session with the sequential search starting from a binary matrix $\left[10;01;00\right]$ in the order of $v_{1}\to v_{2}\to v_{3}$, and Figure 15b shows a session with the cost-sensitivity-based search scheme from the same binary matrix in the order of $v_{3}\to v_{1}\to v_{2}$ in the case of $S\left(v_{3}\right)>S\left(v_{1}\right)>S\left(v_{2}\right)$. In each session, the sensitivity values for the optimization variables were calculated and passed to the next session to determine the search order.

In this study, during the optimization process, a set of candidate joint angle variables was fed into the humanoid model, which simulated a 3D pose. The objective was to determine the joint angle values that minimized the Euclidean distance between the coordinates of each simulated joint and the corresponding measured joints.

**Figure 16:**
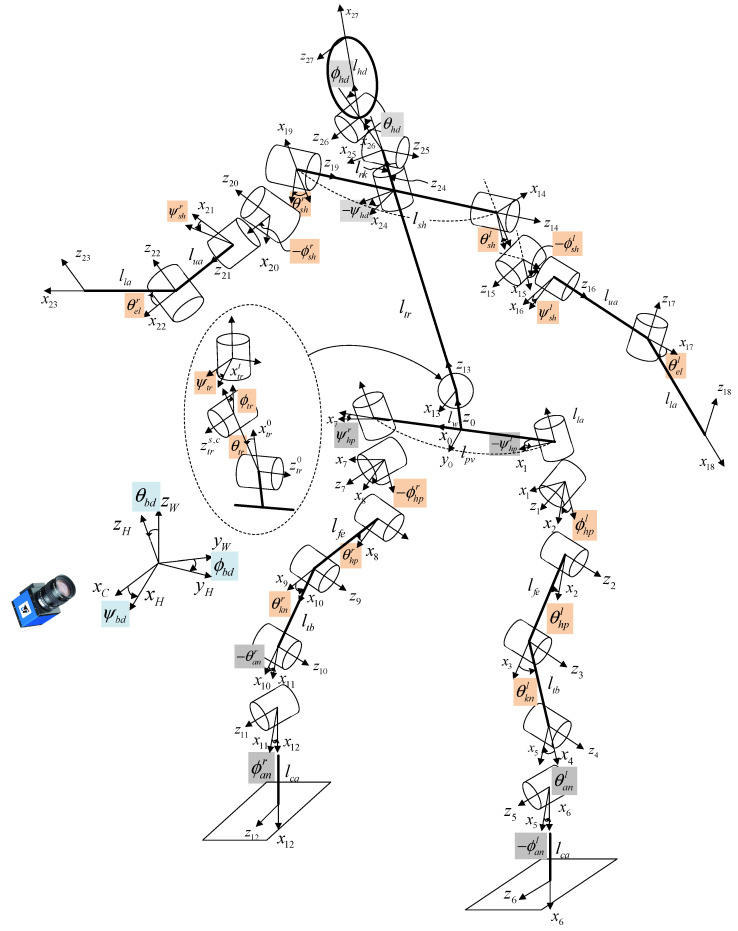
The 3D humanoid robot model with 26 DoF.

The humanoid model has a total of 26 degrees of freedom (DoF), including transversal shoulder joints and a coronal neck joint, as shown in Figure 16, compared with the recent model in [20], where the humanoid model was described with links and joints based on the Denavit–Hartenberg (DH) method [32] with the origin of the reference frame located at the center of the body to create arbitrary poses. Then, 3 DoF lumbar spine joints were added at the center of the pelvis to realize separate upper body motions, and the rotational polarity of all joint variables was defined following Vicon motion capture system [33]. In the figure, the shaded orange variables represent the 17 joint angles used for HPE, and the 3 variables, $\theta_{bd}$, $\phi_{bd}$, and $\psi_{bd}$, are the body angle values related to the relative camera view angle, where θ, ϕ, and ψ denote joint angles rotating on the sagittal, coronal, and transverse planes, respectively. To estimate the arbitrary poses at any distance from the camera, a size factor, γ, is necessary, which is multiplied by each link length. Thus, as the camera moves away from an individual, γ decreases, and vice versa. Therefore, the complete optimization vector for pose estimation comprised the following 21 variables:

(7) $$V={\left[\gamma ,\theta_{bd},\phi_{bd},\psi_{bd},\theta_{ws},\phi_{ws},\psi_{ws},\theta_{hp}^{l},\theta_{kn}^{l},\theta_{hp}^{r},\theta_{kn}^{r},\theta_{sh}^{l},\theta_{el}^{l},\theta_{sh}^{r},\theta_{el}^{r},\phi_{hp}^{l},\phi_{hp}^{r},\phi_{sh}^{l},\phi_{sh}^{r},\psi_{sh}^{l},\psi_{sh}^{r}\right]}^{T}$$

where the superscripts *l* and *r* represent left and right, respectively, while the subscripts *bd*, *ws*, *hp*, *kn*, *sh*, and *el* denote body, waist, hip, knee, shoulder, and elbow, respectively The cost function to be minimized by uDEAS was designed to minimize the mean per joint position error (MPJPE) for the 3D estimated and fitted models and was calculated as the mean Euclidean distance between the 12 joint coordinates estimated by MPP, HybrIK, MHFormer, and D3DP, $\left(x_{e}^{i,j},y_{e}^{i,j},z_{e}^{i,j}\right)$ and those fitted by the 3D humanoid model in Figure 16, $\left(x_{s}^{i,j},y_{s}^{i,j},z_{s}^{i,j}\right)$:

(8) $$L\left(V\right)=\frac{\sum {\left\|\left(x_{e}^{i,j},y_{e}^{i,j},z_{e}^{i,j}\right)-\left(x_{s}^{i,j},y_{s}^{i,j},z_{s}^{i,j}\right)\right\|}_{2}}{12},\;i=l,r,\;j=sh,el,wr,hp,kn,an$$

where the superscripts *l* and *r* represent left and right, respectively, while *wr* and *an* denote wrist and ankle, respectively. When the two models overlap exactly, this value is reduced to zero.

### 3.4. Proposed Method

**Figure 17:**
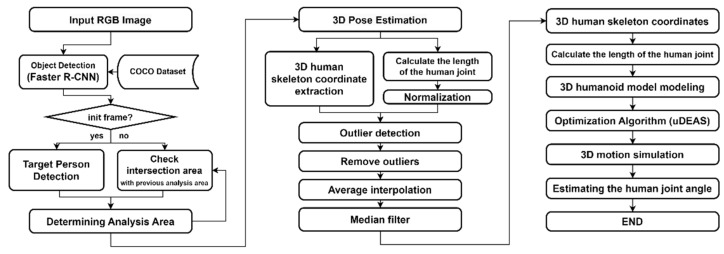
Structure of the proposed 3D pose-estimation algorithm.

In this study, we aimed to improve the accuracy of human joint angle estimation through the aforementioned data-processing steps and the application of a humanoid model using an optimization algorithm to estimate accurate joint angles. The algorithm proposed in this study comprised three major steps, as outlined in Figure 17.

First, the algorithm detected the analysis region in the image data captured using a monocular camera. This involved detecting the person of interest in an RGB image using BBs. Using information from the analysis region of the previous frame, the same person could be tracked continuously and stably. Next, the 3D human joint coordinates were extracted using MPP, MHFormer, and D3DP based on the skeleton model or HybrIK based on the volumetric model. In the next step, outliers that may occur in the DL model were corrected. Here, outliers refer to the jitter in 3D human skeletal coordinates caused by errors in the DL model, misrecognition in occluded areas, and left or right inversion owing to changes in the lighting and clothing patterns. These were addressed through the detection of nonlinear changes in joint length in the human body. Finally, the 3D human skeletal coordinates processed through the correction procedure were reconstructed into a humanoid model using the uDEAS optimization method, which enabled the estimation of the joint angles.

## 4. Experiment

In this study, we evaluated the performance of the four HPE methods by conducting three experiments. First, we checked the number of outliers that occurred in real-world environment video data using the proposed outlier detection algorithm. Table 2 lists the ratio of outliers detected by the DL algorithms for the real-world environment videos. MPP produced the most frequent outliers in real-world environments, and MHFormer exhibited robust performance in joint detection, even in situations similar to real-world environments. At least one outlier was identified in all the DL algorithms.

Next, we compared the computational speed of each HPE method. This study aimed to confirm the applicability of each algorithm in real-time environments. Table 3 lists the average execution times of four HPE methods measured while processing the standing rowing exercise motion. The computational speed measurements were made on the hardware presented in Table 1. Consequently, HybrIK, MHFormer, and D3DP were 7.63, 4.76, and 4.29 times slower than MPP, respectively. Thus, DL-based methods are unsuitable for application in real-time systems in their current state.

**Figure 18:**
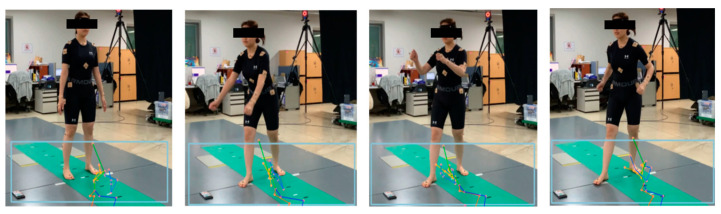
Images captured from a standing rowing exercise (motion 1) video, along with BBs generated by the original HybrIK for HPE (highlighted in light blue).

Finally, we compared the joint angle measured using the Vicon measuring equipment in a laboratory environment with the joint angle calculated using the proposed algorithm. Figure 18 shows the process of analyzing a video shot from the side of a subject performing free gymnastics, similar to rowing, in a motion capture lab equipped with Vicon equipment. In the video, the light-blue boxes represent the BBs that detected the area of a person using the HybrIK’s original code, mistakenly identifying the area from below the knees to the floor as the presence of a person. Consequently, significant errors were observed in the HPE results. This suggests that, as described in Section 3.3.1, HybrIK detects the largest recognized object, which can mis-detect nonhuman external objects.

**Figure 19:**
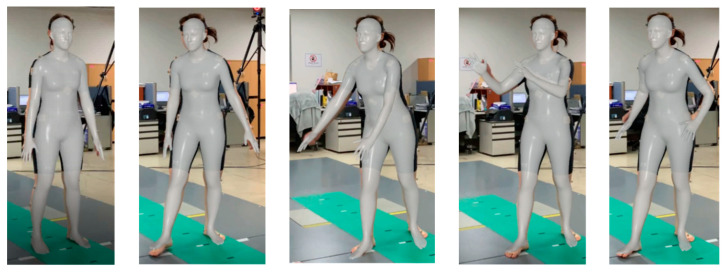
HPE results using HybrIK after the proposed preprocessing of five RGB images from the same video in Figure 18.

Figure 19 shows the results of pose recognition using HybrIK following the application of the proposed filtering algorithm to the RGB images. As shown in Figure 19, it accurately recognized the human poses in the videos.

**Figure 20:**
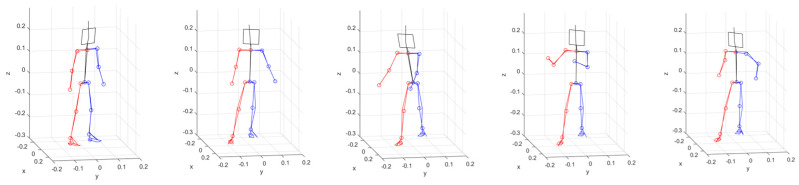
Comparison of measured HybrIK poses (red line: right parts, blue line: left parts, black line: head and torso) and body reconstruction results attained by calculating each joint angle of the humanoid model with uDEAS using the 3D joint coordinate values recognized by HybrIK (lines with circles at the joint).

Figure 20 shows the body reconstruction results obtained by estimating each joint angle of the humanoid model shown in Figure 16 with the uDEAS using the 3D joint coordinate values recognized by HybrIK. These were almost identical for each pose in Figure 19.

**Figure 21:**
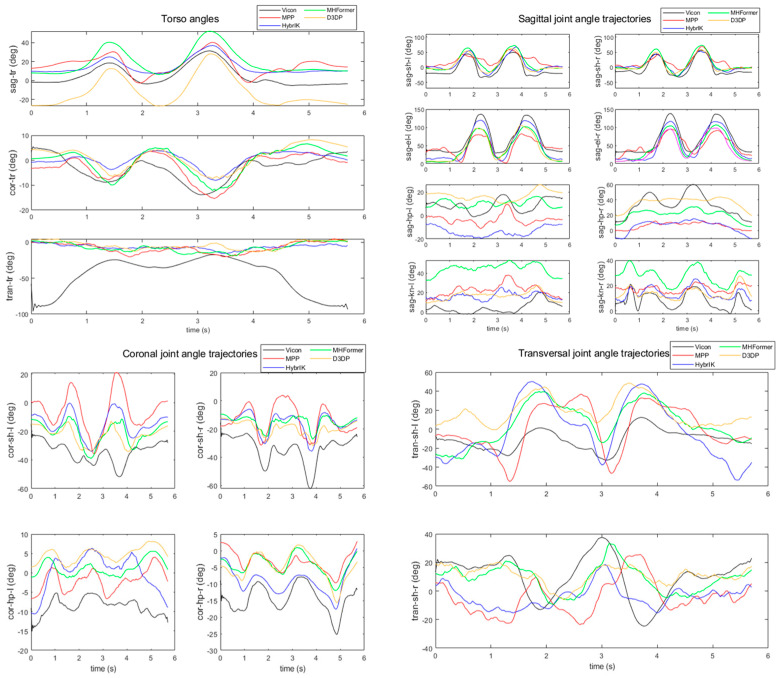
Joint angle profiles calculated by uDEAS from the HPE results attained by modified versions of MPP (red line), HybrIK (blue line), MHFormer (green line), D3DP (yellow line), and Vicon angles (black line).

Figure 21 presents a comparison of the joint angle profiles attained by uDEAS using the joint coordinates estimated by the treated MPP, HybrIK, MHFormer, and D3DP during the standing rowing action. The estimation results for the joint angle profiles of the torso’s sagittal or coronal joint angle, left or right sagittal joint angles of the shoulder or elbow, sagittal right knee joint, and coronal right hip joint exhibited shapes similar to the results obtained using the Vicon system. Similar patterns of angle profiles were observed in the other joints, albeit with certain differences in the offsets.

**Figure 22:**
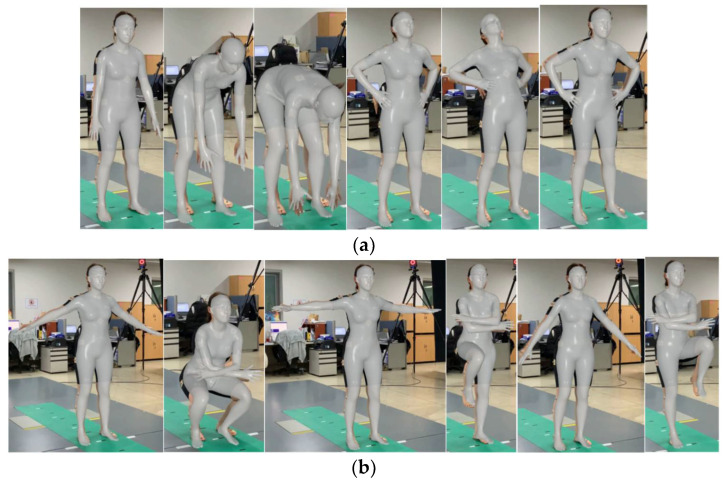
Images captured from (**a**) the back and chest exercise (motion 2) and (**b**) the arm and leg exercise (motion 3) videos generated by HybrIK.

To check the generality, we applied the proposed method to two bare-handed gymnastic movements. Figure 22 shows images captured during the back and chest exercises as the second motion and the arm and leg exercises as the third motion. These movements are suitable for pose recognition and joint angle analysis because they create dynamic poses, such as rotating all joints of the arms and legs and bending or tilting the upper body.

Table 4 lists the mean absolute joint angle error (MAJAE) between estimated and measured angles with Vicon system and degrees of improvement for the three gymnastics exercises using the original MPP, HybrIK, MHFormer, and D3DP and their modified versions proposed in this study. As seen in the Avg. MPJPE column, all human poses matched well with the human simulator’s poses with a maximum joint deviation of less than 4 cm. Among the torso, sagittal (pitch), coronal (roll), and transversal (yaw) joint angles, the torso angles were estimated most accurately, with the average MAJAE being the smallest at 12.14% and the improvement in the average MAJAE being the highest at 35.44% in the sagittal joint angles.

For the coronal joints, it is also encouraging that their MAJAEs were reduced by 27.68% following the application of our treatment algorithm. The transverse joint angles of the shoulder had the largest MAJAE with Vicon data and the smallest improvement effect because the hand shape must also be recognized for accurate measurement; however, we did not measure the hand shape. The overall MAJAE for the three gymnastic motions with the four 3D HPE methods was reduced by 18.99% following the application of the proposed improvement algorithm for human BB recognition and an outlier-correction scheme. We believe that this result is meaningful in that when applying 3D HPE methods to joint angle estimation for HAR, good preprocessing and postprocessing of 3D HPE data can additionally improve the joint angle estimation accuracy. Moreover, this improvement will occur regardless of the 3D HPE method.

## 5. Conclusions

In this study, the limitations of HPE on real-world images were identified, and a method to improve the estimation accuracy was proposed. First, four representative 3D HPE methods, MPP, HybrIK, MHFormer, and D3DP, were introduced, and real-world videos were applied to show the limitations of DL models due to their performance in dealing with the uniqueness of postures and occlusion due to the presence of obstacles, the effects of distance and angle between camera and person, and the effects of light intensity changes due to shadows.

Secondly, signal-processing solutions were then proposed to detect and interpolate jitter, switching, and false-positives by utilizing link length derivatives, mean interpolation, and median filtering to improve estimation accuracy.

Finally, for joint angle estimation using recognized joint coordinates, we applied a more sophisticated 3D humanoid model than the authors’ previous version [20] and a fast optimization algorithm, uDEAS. To investigate the feasibility of real-time pose analysis based on joint angles, we measured the execution time of each HPE and compared the joint angle estimation results for three different motions measured by Vicon.

The proposed pose correction and joint angle estimation approach yielded an overall MAJAE reduction of 18.99%. In addition, HybrIK exhibited an improvement of 61.18% after the proposed improvement algorithm was applied. However, HybrIK exhibited the slowest performance in terms of computational speed. MPP was best suited for applications in real-time environments with a computational speed of 0.0409 s per frame. However, inaccuracies in depth perception and the frequent occurrence of outliers require more attention. Additionally, D3DP and MHFormer showed relatively faster computation speeds compared to HybrIK; however, they encounter difficulties in real-time applications. Moreover, HybrIK and D3DP exhibited the highest accuracies. Although this study corrected anomalies using simple data-treatment methods, future research involving anomaly correction through behavioral analysis will enhance its applicability. Furthermore, joint-angle-based HAR is expected to identify injury risks and dysfunction through gait pattern and exercise motion analyses in the field of sports medicine.

## Figures and Tables

**Table 1:** System device specifications and implementation environment.

Components	Specification
OS	Ubuntu 20.04
CPU	11th Gen Intel Core (TM) i7-11700 @ 2.50 GHz Title 3 (Intel, Santa Clara, CA, USA)
RAM	32.0 GB
GPU	NVIDIA GeForce RTX 3060 (NVIDIA, Santa Clara, CA, USA)
Language	Python 3.8

**Table 2:** Ratio of outliers such as misrecognition in occluded areas and left or right inversion by video file.

Motion	MPP [5]	HybrIK [34]	MHFormer [35]	D3DP [36]
Baseball	10.60%	6.55%	6.17%	5.59%
Cycling	4.12%	100%	5.15%	21.65%
Walking	21.97%	3.79%	2.27%	1.52%
Yoga	2.38%	2.38%	2.86%	1.90%

**Table 3:** Average execution times for the HPE methods using the video file in seconds per frame.

MPP [5]	HybrIK [34]	MHFormer [35]	D3DP [36]
0.0409	0.3120	0.1946	0.1756

**Table 4:** Mean absolute joint angle error (MAJAE) between estimated joint angles and those measured with Vicon system and degrees of improvement for the three gymnastics motions using the original (orig.) MPP, HybrIK, MHFormer, and D3DP and their modified (mod.) versions proposed in this study. The unit of all MAJAE values is degree (Significant results are highlighted underlined).

Motion	Methods	Avg. MPJPE (m)	Torso MAJAE (3 Joints)	Sagittal MAJAE (8 Joints)	Coronal MAJAE (4 Joints)	Transversal MAJAE (2 Joints)	Avg. MAJAE	Improvement
**1** **(standing rowing exercise)**	**MPP (orig.)**	0.0160	18.80	19.66	16.71	21.11	18.99	1.53%
	**MPP (mod.)**	0.0160	18.39	19.18	16.74	21.13	18.70	
	**HybrIK (orig.)**	0.0145	43.31	49.99	37.60	34.31	44.05	62.56%
	**HybrIK (mod.)**	0.0045	18.04	16.81	12.82	20.24	16.49	
	**MHFormer (orig.)**	0.0349	18.91	20.86	13.02	14.66	17.94	2.20%
	**MHFormer (mod.)**	0.0346	19.13	19.78	13.11	15.08	17.55	
	**D3DP (orig.)**	0.0348	22.82	16.72	11.91	19.71	17.02	5.40%
	**D3DP (mod.)**	0.0332	21.44	15.08	12.27	19.79	16.10	
**2** **(back and chest exercise)**	**MPP (orig.)**	0.0205	9.33	20.66	22.31	33.99	20.62	0.57%
	**MPP (mod.)**	0.0205	8.96	20.31	22.60	34.37	20.50	
	**HybrIK (orig.)**	0.0153	21.16	52.92	41.79	44.44	43.70	56.11%
	**HybrIK (mod.)**	0.0064	10.15	18.33	19.77	34.96	19.18	
	**MHFormer (orig.)**	0.0258	10.65	19.89	19.83	25.66	18.92	1.35%
	**MHFormer (mod.)**	0.0252	10.39	19.40	19.98	25.52	18.67	
	**D3DP (orig.)**	0.0350	12.85	16.27	18.96	18.78	16.59	7.27%
	**D3DP (mod.)**	0.0265	8.42	15.42	18.98	18.51	15.39	
**3** **(arm and leg exercise)**	**MPP (orig.)**	0.0209	7.17	17.65	16.47	24.76	16.36	5.74%
	**MPP (mod.)**	0.0198	7.24	16.24	15.64	23.98	15.42	
	**HybrIK (orig.)**	0.0163	24.57	50.72	37.60	36.04	41.29	64.87%
	**HybrIK (mod.)**	0.0051	6.90	15.69	12.36	25.48	14.51	
	**MHFormer (orig.)**	0.0343	7.49	20.61	16.32	46.56	20.34	10.74%
	**MHFormer (mod.)**	0.0336	7.51	17.90	14.66	42.13	18.16	
	**D3DP (orig.)**	0.0325	9.18	16.59	16.30	40.62	18.04	9.60%
	**D3DP (mod.)**	0.0318	9.13	14.08	15.48	37.65	16.31	
**Avg. MAJAE (orig.)**			17.19	26.88	22.40	30.05	24.49	18.99%
**Avg. MAJAE (mod.)**			12.14	17.35	16.20	26.57	17.25	
**Improvement**			29.35%	35.44%	27.68%	11.59%		

## Data Availability

The raw data supporting the conclusions of this article will be made available by the authors on request.
